# Emergency Department Capacity Planning: A Recurrent Neural Network and Simulation Approach

**DOI:** 10.1155/2019/4359719

**Published:** 2019-11-15

**Authors:** Serkan Nas, Melik Koyuncu

**Affiliations:** Department of Industrial Engineering, Çukurova University, Sarıçam 01330, Turkey

## Abstract

Emergency departments (EDs) play a vital role in the whole healthcare system as they are the first point of care in hospitals for urgent and critically ill patients. Therefore, effective management of hospital's ED is crucial in improving the quality of the healthcare service. The effectiveness depends on how efficiently the hospital resources are used, particularly under budget constraints. Simulation modeling is one of the best methods to optimize resources and needs inputs such as patients' arrival time, patient's length of stay (LOS), and the route of patients in the ED. This study develops a simulation model to determine the optimum number of beds in an ED by minimizing the patients' LOS. The hospital data are analyzed, and patients' LOS and the route of patients in the ED are determined. To determine patients' arrival times, the features associated with patients' arrivals at ED are identified. Mean arrival rate is used as a feature in addition to climatic and temporal variables. The exhaustive feature-selection method has been used to determine the best subset of the features, and the mean arrival rate is determined as one of the most significant features. This study is executed using the one-year ED arrival data together with five-year (43.824 study hours) ED arrival data to improve the accuracy of predictions. Furthermore, ten different machine learning (ML) algorithms are used utilizing the same best subset of these features. After a tenfold cross-validation experiment, based on mean absolute percentage error (MAPE), the stateful long short-term memory (LSTM) model performed better than other models with an accuracy of 47%, followed by the decision tree and random forest methods. Using the simulation method, the LOS has been minimized by 7% and the number of beds at the ED has been optimized.

## 1. Introduction

Health is the primary asset for human beings, and the health needs of a population in a country are typically met through the healthcare system. In the healthcare service network, the primary healthcare units are hospitals, in which emergency departments (ED) play a critical role. At a hospital's ED, every arriving patient has to be examined which causes high operating costs and results in budget shortages. Moreover, any serious error in the practice of an ED may be life-threatening [1]. Thus, the effectiveness of EDs is crucial in increasing the quality of health services, particularly in developing countries. The effectiveness of EDs depends heavily upon the allocation of hospital's critical resources such as space and budget.

Our observation on healthcare systems is that there is no strong correlation between the health expenditure and the effectiveness of the healthcare systems. This implies that optimizing the resource allocation has a critical role in increasing the quality of healthcare systems, particularly in developing countries with limited resource capacity. In the improvement of the resource planning and the management of hospitals, various operational research/management methods are employed including mathematical programming, queuing theory, and simulation. These methods are more needed when EDs face overcrowding and high uncertainty. Obviously, the fact that patients cannot make an appointment for ED service causes uncertainty. The uncertainty of patients arriving at EDs causes overcrowding, thereby resulting in more waiting time and less healthcare service quality. One should note that EDs face uncertainty not only in terms of patient arrivals, but also in terms of the treatment times.

Simulation is one of the most effective methods used for modeling the systems with a high degree of uncertainty. Therefore, simulation is an important method that contributes to the operational, tactical, and strategic decision-making process about EDs [2]. The success of the simulation model depends on its resemblance to the corresponding real system as much as possible, and to achieve this, the ED simulation model needs an accurate estimation of patient arrivals and the length of stay (LOS). Therefore, the prediction of patient arrivals has a vital role in modeling the system. In addition, the most significant cause of ED crowding is the inability to manage inpatient bed availability, thereby making it critical for hospitals to manage patient flow [3]. Human resource allocation is also adjusted according to the bed demand to treat patients in the ED with the aim of providing universal access to healthcare to all the patients therein [4]. Predicting patients' arrivals rates and, consequently, the bed demand at EDs may lead to effective use of hospital resources and reduce the overcrowding and waiting time of the patients at ED [5]. There are different approaches to predicting patient arrival rates. Machine learning as a subset of artificial intelligence (AI) is one of the best approaches in the prediction of target variables such as patient arrivals. AI technologies are likely to impact many aspects of emergency medicine in the near future [6]. Apart from the traditional mathematical models (linear or nonlinear), the artificial neural network (ANN) approach (which is a set of algorithms used in ML) can handle complicated problems associated with a large number of datasets; further, the implemented ANN is user friendly for the hospital staff, as a nonsite predictive tool [7].

Published literature associated with modeling the visits of patient to EDs, in general, use two types of methods. The first method analyzes the correlations that are often linear between patient arrivals and a number of independent variables, such as calendar or weather variables; the second method predicts future values from the past values using the assumption that patient visits obey a time series [8]. In the literature, the features are generally temporal, weather, or demographic variables. Furthermore, the literature studies generally agree on the point that temporal variables are more important than weather variables for forecasting the patient arrival rates at EDs. All these studies used any subset of these variables. The patient arrival problems are generally related daily [7, 9–14], weekly [4], or monthly [4, 15]; however, a few studies focus on hourly arrival rates which include high variation [16–18]. The forecasting accuracy worsened as the forecast time intervals became smaller: the best MAPE rate is 2% for a month, 11% for a day, 38% for four-hour period, and 50% for an hour [18]. These results of forecasting accuracy are very close to those in the other studies in the literature, with the variation mainly due to the used dataset and prediction algorithms. The studies were performed using different types of patient data, such as one-year data [4, 5, 9–11, 16, 17], 2- to 6-year data [7, 12–15, 17], 10-year data [19], and the daily number of patient arrivals generally used over a period ranging from 1- to 10-year data (usually 3 years) [8]. Different techniques used for the prediction of patient arrival rate are the ANN [7, 9, 11, 14], the autoregressive integrated moving average (ARIMA) [4, 8, 12–14], the linear regression (LR) [14, 18], the exponential smoothing [14, 15, 18], the logistic regression [5, 10, 11, 19], the decision tree (DT) [1, 10], the gradient boosted machines (GBM) [10], the Poisson regression model [16], the random forest (RF), the AdaBoost (AB), the support vector machine (SVM) [5], and the LSTM model [20].

In this study, nine different ML techniques and the stateful LSTM model have been used to predict patient arrival rates. The LSTM model is a type of recurrent neural network (RNN), which is a class of the ANN. ANNs are used in many ED studies, such as optimum resource planning [1], modeling a ward [7], predicting patients' arrival rate [20], diagnosing illness [21, 22], and predicting patient LOS at EDs [23]. This study is different from the LSTM model [20] by predicting hourly patients' arrival not only forecasting the daily patients' arrival.

It is worthy to point out that the employed algorithm must be assisted by feeding it with appropriate features. Due to the fact that wrongly selected features can decrease the effectiveness of the prediction, feature selection is a critical part of ML and plays a significant role in creating an effective model for ML studies. Intending to achieve this, this study employs two main methods: (1) a wrapper method that evaluates the contribution of features by using the ML algorithm and (2) a filter method that ranks the variables according to the heuristics based on general characteristics of the data and not testing the subset of the features by any ML model to determine whether a feature increases the accuracy of the prediction. Because of its exhaustive nature, the wrapper method has been considered to enhance the model's performance with better feature subsets. When we compare the filter method with the wrapper methods, the latter is slower [24, 25]. The wrapper method that uses correlation coefficients will have faster processing ability and makes a better subset of the features [26]. Another variant of the wrapper method is the exhaustive feature-selection method, which finds the best of the features by minimizing the loss criteria of the chosen algorithm. However, this variant is not suitable for a large number of features, but if computing time can be handled, this is the best method. An exhaustive feature-selection method has been used as this method tests all the combinations of the features and finds the best subset of the features.

**Figure 1:**
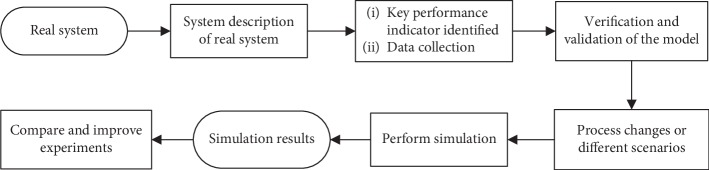
Flowchart of the typical DES model.

There are different types of computer simulation methods such as system dynamics (SD), discrete-event simulation (DES), and agent-based simulation (ABS) models. DES models and SD models have used modeling under different situations. Comparing the DES model with the ABS model regarding which one is better in the simulation of the real-world problem remains controversial [27]. Modeling an ED by using the DES models enables us to analyze the system and how the process changes influence patient flow and resource availability [28]. In this study, the DES model has been chosen, suitable enough for tackling complex systems such as EDs. By changing the number of beds, the reduction in patients' waiting times has been sought using our developed DES model. The procedure of our developed simulation method is depicted in Figure 1.

We have observed some potential gaps in the literature and have tried to contribute. First, the main contribution of this study is to use ML algorithms to generate input parameters to a DES model which is then used to optimize resource allocation in an ED. In particular, we use ML algorithms to predict patient arrivals and use simulation optimization to determine the capacity of the ED (in terms of the number of beds) that leads to the desired patient LOS. This is in contrast to using a theoretical (e.g., Poisson) arrival process or a deterministic arrival pattern which may lead to a significant inaccuracy in prediction and inefficiency in resource allocation. Second, this study predicts the patients' arrival rate and is one of the rare studies that use the LSTM model–a type of RNN–in doing so. Third, this study identifies the best subset of the features by using the exhaustive feature-selection method which has not been used in the previous studies in prediction of patient arrival rates. Moreover, few applications are found in hourly patient arrival problems, and the studies used therein [16–18] usually use the data for one year; however, this study uses real data, hourly one year's data, daily data, and also five years' hourly data to check if the additional data improve the accuracy of the prediction which yields positive results.

## 2. Methods

**Figure 2:**
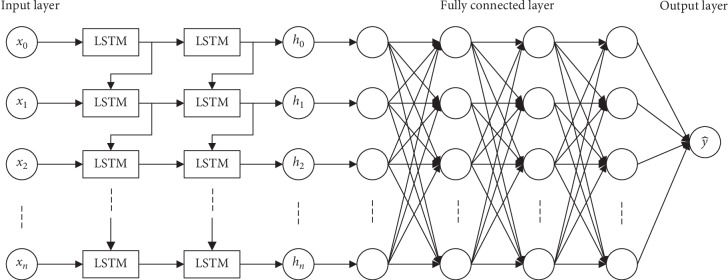
Structure of the stateful LSTM model.

This study is designed in four sections to contribute to the relevant literature. First, the system has been analyzed and the overall system structure has been described for the simulation model. Second, all the required data have been collected, and the best subset is found by using the exhaustive feature-selection method. Third, an appropriate RNN model is chosen, and then, the parameters of the model are selected, following, the stateful LSTM model, which is a type of RNN model, has been constructed. The structure of the stateful LSTM model is presented in Figure 2. Also the other 9 ML algorithms are determined and their models have been developed by using Python programming. Patients' arrival rates have been estimated by using 10 ML techniques including the stateful LSTM models. All the strategies, comparisons, and adjustments have been executed at this stage to predict the most accurate hourly and daily patient arrival rates. Fourth, the simulation model has been designed according to the system description, after which the verification and validation of the system have been performed. By changing the number of beds, the minimization in the patients' LOS and the optimization in the number of beds have been sought. Hourly patient arrival rates, which are the outcomes of ML algorithms, have been used as the simulation input variables. The duration of treatments and the hospital services have been taken from the observations determined by the hospital management; subjective discretion has been used. The patient route in ED acts as the third input extracted from the analysis of hospital data. The details of each section are as follows.

### 2.1. Outcome Measures: Hourly Patient Arrival and Optimum Number of Beds

The first outcome for the analysis is the total number of hourly ED arrivals for patients. Some studies categorize the patients and drop some of them according to their constraints. Because this input (i.e., the total number of hourly ED arrivals) is used for the simulation to allocate bed resources, categorizing and then eliminating may give inadequate results. Hence, all the patient arrivals have been included in this study. The second outcome is the optimum number of beds, obtained by using our developed simulation model.

### 2.2. Section One: System Description

**Figure 3:**
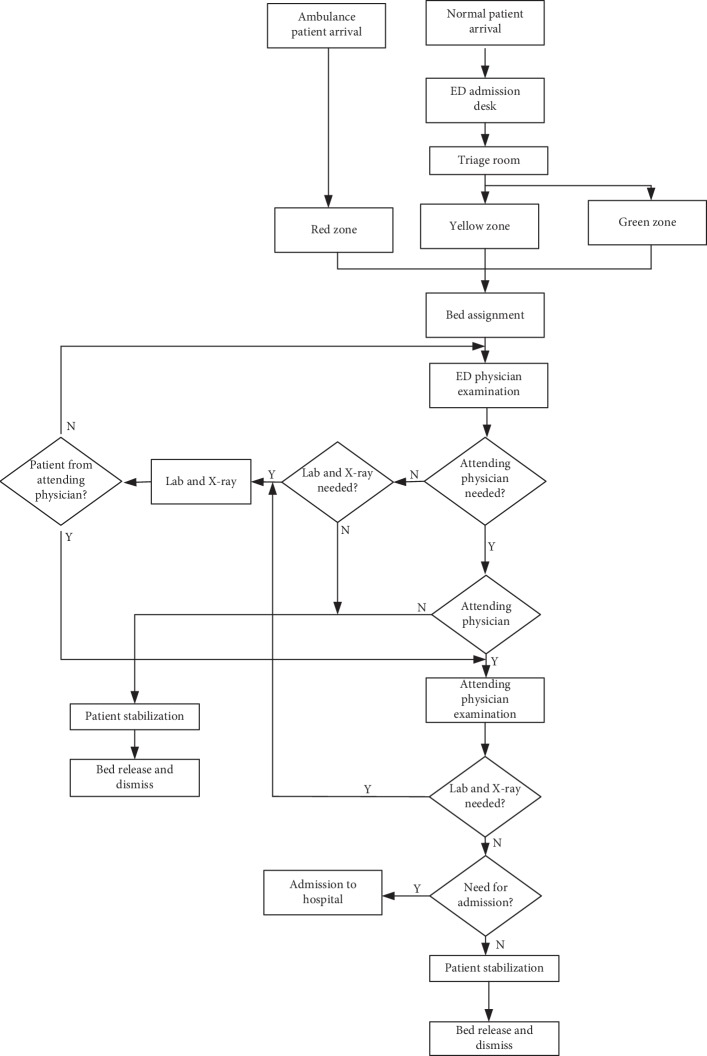
Flow of patients at the emergency department.

The private Ceyhan Çınar Hospital is the first and only private hospital in the Ceyhan district of Adana in Turkey. The ED therein operates 24/7 and receives 75 patients per day on average. The X-ray facility and the laboratory are very close to the ED. The ED process starts with the arrival of patients. Patients may arrive at the ED by their own or by ambulance. Except the patients arriving by ambulance, all the patients must visit the receptionist to register their personal information, and then, the triage nurse checks the acuity of the patient on the basis of their triage system. According to the triage system of this hospital, the patients are categorized into three categories: red, yellow, or green. The red class is for the patients that need the most urgent treatment, the yellow for normal acuity, and the green for the lowest acuity. Following the triage process, the patients are treated by the emergency physician. If needed, the emergency physician refers a patient to the laboratory and the X-ray facility. After further examination, the patient should go to the patient stabilization room and, if necessary, the emergency physician can refer the patient to an attending physician. The attending physician further may refer the patient again to the laboratory or the X-ray facility. After the examination, the attending physician can refer the patients to hospital admission or the stabilization room and then discharge the patients from the ED. Anyhow, patients arriving by ambulance can be excused from the registration process at the triage room, and they directly go to the emergency physician. Except this part, the process is the same as that with the other patients. The flow of the patients is demonstrated in Figure 3.

### 2.3. Section Two: Data Collection and Feature Selection

#### 2.3.1. Data Collection

This study focuses on the number of patient arrivals, which span across a total of 8760 hours in the one-year study period (January 1, 2017–December 31, 2017) because it aims to predict the hourly arrival rate to the ED with consideration that the analysis of daily data rather than hourly would lead to loss of significant information regarding variant patient arrivals. We classified the arrival date for each study hour on the basis of the calendar year, season, weekday, weekend, and hour of the day. Additionally, we have identified all the holidays, whether official or religious and assigned additional information to the hourly data whether it is a holiday or a normal day. We defined the seasons as winter (December 1, 2017–January 1, 2017, and January 1, 2017–February 28, 2017), spring (March 1, 2017–May 31, 2017), summer (June 1, 2017–August 31, 2017), and fall (September 1, 2017–November 30, 2017). However, we collected the data of the weather every three hours (i.e., 3 am, 6 am, and 9 am) and not every hour. So, for the hours during which we could not reach the weather data, we assigned the data to two hours after the time on which we had reached the data. For example, if the data were collected at 3 am, then we assigned the data collected at 3 am to the weather data at 4 am and 5 am. We performed the same procedure for the other four years, i.e., 2016, 2015, 2014, and 2013. Also, for the year 2017, we prepared the daily arrival rates by using the same features except the hour of the day and the mean arrival rate.

#### 2.3.2. Feature Selection

**Figure 4:**
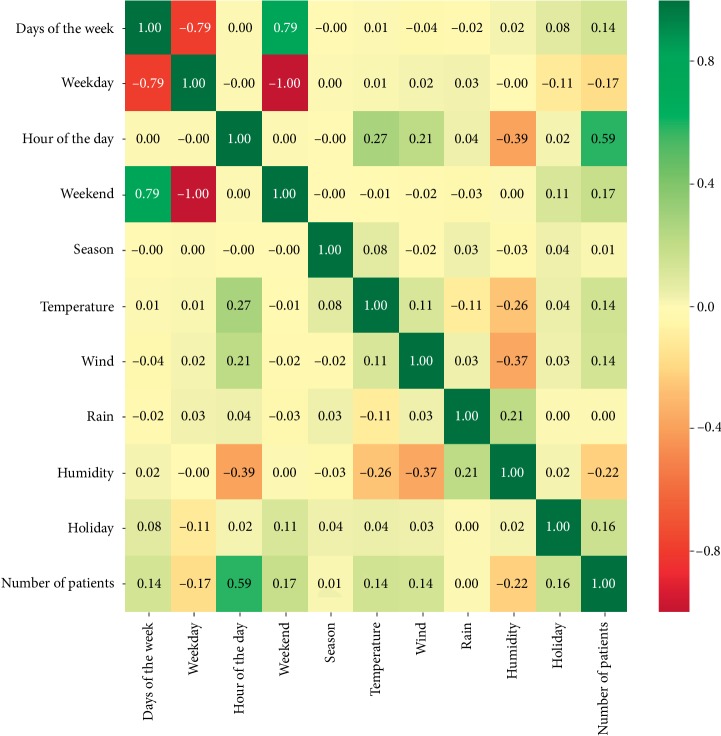
Correlation matrix of the features and the number of patients.

We needed the future selection process to reduce overfitting and training time and also to improve accuracy. We determined the features on the basis of the previously conducted studies [1, 4, 5, 8, 10–12, 14, 16, 18, 19] and the data availability. After the determination of the features, we performed the exhaustive feature-selection method to eliminate any doubt about the features subset because the method selects the optimum subset by minimizing the loss function with the help of any ML techniques and tests all the combination of the features. In this study for evaluating the contribution of features, we applied the RF regression method, and for loss function, we employed the mean absolute error (MAE) method which is described in Section 2.4.2. Also, we calculated the correlation values, as shown in Figure 4 and determined that the day of the hour has a moderate positive relationship with the number of patients and also this table will help us in comparing with the correlated variables and the variables of best subset of the features.

### 2.4. Section Three: Machine Learning Algorithms and Stateful LSTM Method

#### 2.4.1. Machine Learning Algorithms

ML, a subset of artificial intelligence, focuses on learning for themselves. The prediction task is executed using ML algorithms which are defined as learning a target function (*f*) that best maps input variables (*X*) to an output variable (*Y*): *Y* = *f*(*X*). A neural network, which is an ML algorithm, computes systems on the basis of the neural structure of the brain. Unlike the human brain, the integrated circuits are two-dimensional devices consisting of layers. The layers can be at least three: first for input, the last for output, and the hidden layers between the input and output layers to compute and solve the real problem. Neurons convert weighted input to its output that passes to other neurons by using the activation function. The supervised learning, which is a task of interfering a function from labeled training data, is one of the best ways to predict patient arrival rates. The best features and the best ML algorithm are vital to predicting unknown test sets with high accuracy. The trial-and-error approach could help us choose the best learning algorithm. Consequently, we tried different algorithms' performances and selected the winner for our problem. Except the stateful LSTM method, nine ML algorithms that were applied to the training data to build the models are as follows: DT, SVM, GBM, AB, stochastic gradient regression (SG), K-nearest neighbors (KNN), RF, multilayer perceptron (MLP), and LR.

#### 2.4.2. Constructing the Architecture of a Type of RNN, Stateful LSTM, Forecast Accuracy Measures

RNNs are an advanced type of ANNs, and unlike the traditional neural networks, RNNs can learn to use the past information. RNNs are popular models, which have shown a great promise in many tasks. They do not assume that inputs are independent of one another and use sequential information. Whenever there is a sequence of data and the temporal dynamics that connects the data are more important than the spatial content of each frame, one needs to use RNNs. RNNs connect to the past data to the present one, thereby allowing the information to persist if the past information is the close to the point where it is required; otherwise, RNNs cannot manage connecting the information. However, LSTM networks solve this connection problem as they are capable of learning long-term dependencies. For these reasons, in this study, the LSTM model, which is a very special kind of RNNs, has been used and is found out to be much better than the standard version. The correlation coefficient, between the features and the number of patients, was calculated and is presented in Figure 4, which shows that the hour of the day is highly correlated with the number of patients. This correlation shows us that arrival rate is sequential dependent. Because the arrival rate to EDs is sequential dependent, we designed an RNN, which is a model type of ANN. Also, the type of ANN used is significant for ensuring the accuracy of the model. The accuracy of ANN models depends upon the chosen parameters, the number of hidden layers, learning rate, etc. By adjusting these parameters, the model either performs better or not. We chose the activation function, the leaky ReLU. As an optimizer, we used the Adam optimization because hourly patient arrival data include a lot of variation and noise and these data are nonstationary data. We tested different numbers of hidden layers and then adjusted the model to 300 hidden layers. Afterward, we tuned the learning rate to 0.0005 and used 80% of the data for the training process and the remaining data for validation. Further, we scaled all the attributes by using min-max scaler to ensure better model performance. We used the loss function MAPE and MAE to improve the model's accuracy because MAPE is scale-independent, which is frequently recommended as the primary measure of forecast accuracy, particularly for comparing forecast models across datasets with different scales [29]. But if any actual data in the dataset are close to zero, then MAPE is undefined or the actual data must not be included in MAPE calculation [30]. In addition, considering MAPE for resource allocation guides the model in a right way. For a series of predicted values (*p*_1_, *p*_2_,…, *p*_*n*_) and the corresponding series of actual values (*x*_1_, *x*_2_,…, *x*_*n*_), we have the following equations:

(1) $$\begin{array}{c} \text{MAPE}=\frac{100\%}{n}\overset{n}{\underset{i=1}{\sum }}\frac{\text{abs}\left(x_{i}-p_{i}\right)}{x_{i}},\\ \text{MAE}=\frac{1}{n}\overset{n}{\underset{i=1}{\sum }}\text{abs}\left(x_{i}-p_{i}\right). \end{array}$$

We tried to find the best parameters for our model using the trial-and-error method, but finding the best parameters for any project still remains an open question.

### 2.5. Section Four: Simulation Model

**Figure 5:**
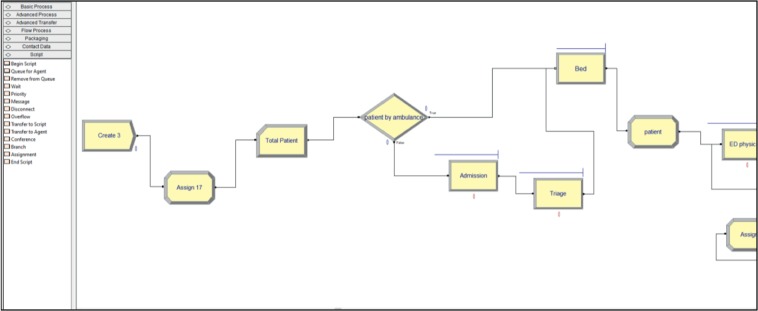
User interface from Arena software.

We developed a discrete-event simulation model to analyze the ED's process. We use the Arena, which is one of the most popular software for DES. Arena is a true Microsoft Windows operating system application, so most features and operations existed in it are familiar to the end users. Arena allows to integration with Microsoft Technologies such as importing Microsoft Visio flowcharts as well as reading the data from or sending to the outputs to Excel [31].  Figure 5 shows a screenshot of the user interface for part of our developed model.

In order to get reliable results from simulation models, accurate data are essential. Therefore, we predicted the most important data, hourly patient arrival rates, by 10 ML algorithms. Further, we used the most accurate result as an input for our developed simulation model. The other required data are the treatment time of each doctor and the percentage number of all the patients for each route followed by patients in the ED. We made observations and took the advice of the hospital management to determine the treatment and hospital service time. We determined the percentage number of each route by using the process of data analysis. The analysis revealed that 70% of the patients coming to ED are sent to the laboratory and X-ray sections and nearly half of the patients coming to ED are sent to the attendant physician. The emergency physician sent 22% of the patients to a pediatrician, 17% to an otorhinolaryngologist, 13% to an internal medicine specialist, 11% to an orthopedics and traumatology specialist, and the rest to ten different attending physicians. After the treatment, all of the patients are sent to the stabilization room. The treatment service times for each section at the ED are presented in Table 1.

#### 2.5.1. Verification and Validation

Simulation model should be as similar as possible to the actual system. To check that the model is created correctly, the hospital management performed verification. The coordinator of the hospital management owns the responsibility to confirm that the simulation model is correctly implemented and that the model is a good representation of the conceptual model.

#### 2.5.2. Determining the Number of Replication

Replicating the simulation model more than once makes the simulation results more reliable and determines the width of the confidence interval. To determine the best replication number, the fixed sample size method or the sequential method is generally used. In this study, we use the sequential method to determine the replication number. This method leads to a sequence of steps [32]. Davis et al. also used the sequential method to determine the optimal replication number [33].

 
$\bar{X}\left(n\right)$: sample mean
 
*δ*(*n*, *a*): confidence interval half length
 
*s*^2^(*n*): sample variance
 
∝: significance level
 
*t*_*i*−1,1−*α*/2_: *t* table value
 
*γ*0′: *δ*(*n*, *a*) = $t_{i-1,1-\alpha /2}\sqrt{\left(s^{2}\left(n\right)\right)/n}$

The steps of the evaluation procedure are as follows.

The starting number of the replication must be chosen as *n*_0_  ≥ 2 and *n* = *n*_0_.

1. Compute $\bar{X}\;\left(n\right)$ and *δ*(*n*, *a*) from *X*_1_, *X*_2_,…, *X*_*n*_.
2. If $\delta \left(n,a\right)/\bar{X}\left(n\right)$ ≤ *γ*′, use $\bar{X}\;\left(n\right)$ as the point estimate for *μ* and stop. At this point; *I*(*α*, *γ*) = $\left[\left.\bar{X}\left(n\right)-\delta \left(n,a\right),\bar{X}\left(n\right)+\delta \left(n,a\right)\right]\right.$ is an approximate 100(1 − *α*) percent confidence interval for *μ* with the desired precision.
3. Otherwise, replace *n* by *n* + 1, make an additional replication of the simulation, and return to step one.

We used the method mentioned above and found that the number of replication must be 5.

#### 2.5.3. Determining the Warm-Up Period

**Figure 6:**
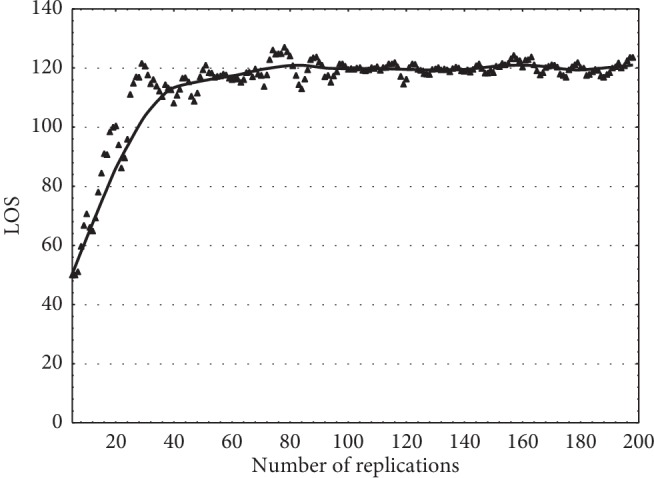
Moving average with *w*=1 for length of stay, ED system.

The simulation must be set to warm-up period to make the simulation reach steady state to eliminate initial bias and achieve reliable results. Kadı et al. [34] used Welch method for determining the warm-up period [34]. To identify the warm-up period, we also used Welch graphical method. According to the Welch graphical method, first we ran the model with the one-time unit of the replication length and then recorded the observed average values of the chosen performance criteria. Second, we determined the time period (*w*) and add to it the past *w* days and the following *w* days of this observed value, and then we take the average of these values and plot them on graph. We repeated this method by increasing one-time unit at every simulation run. When the trend of the observed values came to end and began to smooth, we determined that point as the warm-up period of the simulation model [32]. In the model proposed by us, we determine the time period (*w*) as 1 and set the warm-up period to 29 hours, as shown in Figure 6. To make bias near zero in our simulation results, we ran the model for 7500 hours and with five replications for the 10 different data groups.

**Figure 7:**
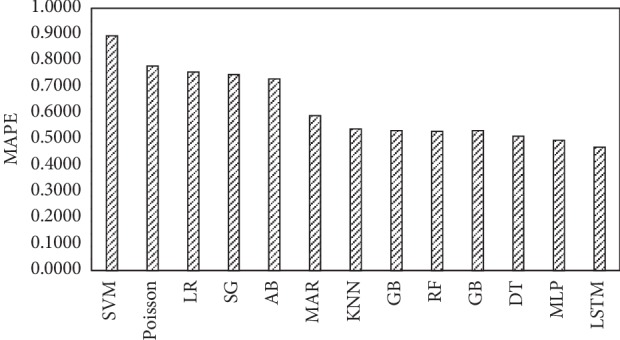
Comparison of the forecast accuracy between the LSTM, Poisson, mean arrival rate, and the other 9 ML algorithms based on MAPE values.

**Figure 8:**
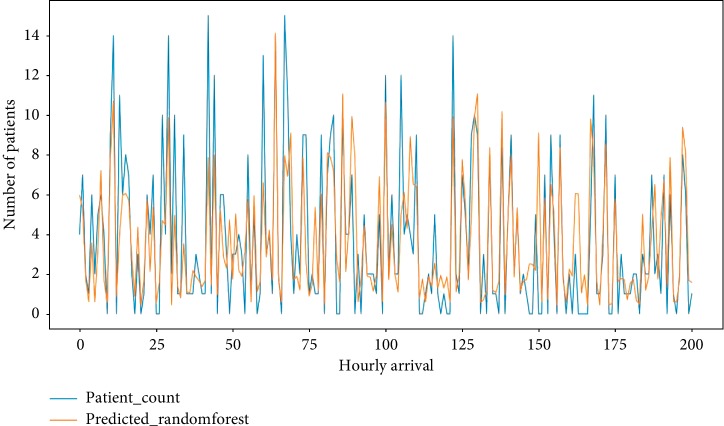
Comparison of the actual number of hourly patient arrivals and the prediction number of hourly patient arrivals to ED.

Figure 7 shows the forecast accuracy (based on MAPE) of Poisson hourly arrival rates, only hourly arrival rates, and the prediction of hourly arrival rates by the stateful LSTM and the other 9 ML algorithms. Figure 7 shows that our simulation model must use the outputs of the stateful LSTM to be a more accurate representation of an actual system. The comparison of the actual number of arrival rates to ED and the number of arrival rates which are predicted by LSTM model is shown in Figure 8. Unfortunately, the Poisson arrival rates have less accuracy (78%) than that of the mean arrival rates (59%). However, most of the reviewed studies used the fact that the patients' arrival rate to an ED is a nonhomogeneous Poisson process with rate *λ*(*t*) [35, 36].

## 3. Results

### 3.1. Statistics of Patient Arrival

Every hospital has some special characteristics. At this hospital, 60% of the patients arrive at the ED between 5 pm and 12 pm, the most crowded time being between 8 pm and 11 pm and the least crowded time between 4 am and 6 am. Further, 36% of the patients come at weekends, and the rest of them come at weekdays. In the winter and summer, the number of patient increases; however, the number of patient decreases by 13% in autumn compared to that in winter. On official holidays, the hourly patient arrival rate to ED is 5.45; however, on normal days, the hourly patient arrival rate is 3.31. On nearly every weekday, the patient arrival rate is the same: daily patient arrival to the ED constitutes 13% of the patients coming to the ED; on weekends, the daily patient arrival to the ED constitutes 19% of patients coming to the ED for each day.

### 3.2. Feature Optimization

Our target is to find the most accurate predictions and then use these predictions as an input to our simulation model to minimize patients' waiting time and optimize the number of beds in EDs. To find the most accurate predictions, we needed to determine the best subset of the features. We calculated the correlation coefficients between all the variables, as shown in Figure 4. The most correlated variables with the number of patients are the mean arrival rate, hour of the day, humidity, and weekday. However, the combination of the features can give very different results. Hence, to find the optimum subset of the features, we used the exhaustive feature-selection method and determined the best subset with mean arrival rate, weekday, season, and holiday as features. This best subset is determined for both the 5-year hourly arrival rate data and the one-year hourly arrival rate data. For daily arrival rate data, we determined the optimal subset of features such as temperature, humidity, days of the week, season, holiday, and weekend.

### 3.3. Model Performance

We used the ML algorithms to predict the patients' arrival rates. First, we predicted the daily arrival rates, as shown in Table 2.

As shown in Table 2, the stateful LSTM method has the lowest MAPE mean and thus gives the best result. However, EDs have a very dynamic structure, and the predictions of arrival rate will be used as an input for the simulation, so the hourly arrival rate is predicted with the 2017 year data, which comprises 8760 hourly patient arrival rates. The prediction of hourly arrivals also offered the possibility of higher administrative response than that while predicting ordinary arrival rates during a several-hour period.  Table 3 presents the performance of ML according to the MAPE of the predictions, and the RF model gives us the best performance.

We aimed at increasing the accuracy of the prediction, so our expectation is that the additional data could potentially increase the accuracy [10]. So we took the data between the years 2013 and 2018 from the hospital management system and adjusted them for use by ML algorithms. By comparing RF mean value from Table 3 with LSTM mean value from Table 4, we calculated the increase in the accuracy of the prediction as 5.9%.

**Figure 9:**
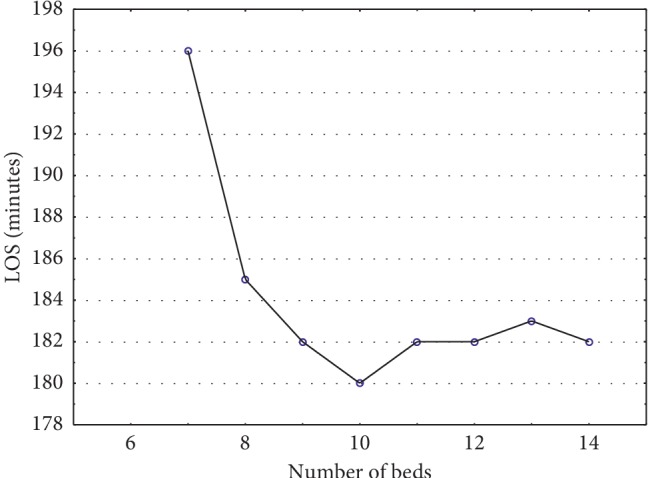
Impact of changing the number of beds on the total length of stay.

**Figure 10:**
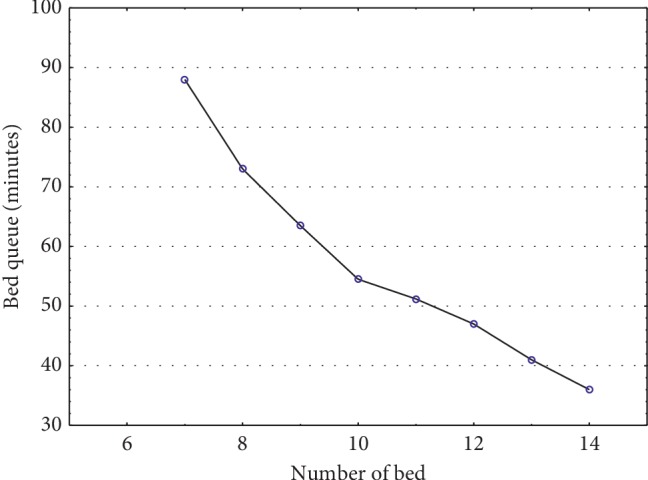
Impact of changing the number of beds on the length of bed queue.

So, we used the output of the predictions, calculated by the stateful LSTM model by using 5-year hourly arrival data, as an input for our developed simulation model to minimize LOS. A lot of factors affect the LOS, but in this study, we focused on the number of beds and its impacts on the LOS. While other resource remains the same, we changed the number of beds and analyzed the LOS by performing the simulation.  Figure 9 depicts the impact on LOS by changing the number of beds, and Figure 10 depicts the impact on bed queues by changing the number of beds. This impact assessment is expected to be helpful for hospital managements for their tactical decision-making process.

## 4. Discussion

In general, empirical knowledge or correlation analyses were performed to select the variables that contribute more to the accuracy of prediction [1]. In this study, in contrast to using the correlation analysis or other feature-selection techniques, the exhaustive feature-selection method was used to find the best subset of features. For example, humidity and temperature were observed to be correlated with the number of patients whereas holiday and season have less correlation, as presented in Figure 4. In contrast, for the hourly arrival rate data, the best subset of the features is as follows: mean arrival rates, weekday, holiday, and season. However, for daily arrival rate data, the best subset of the features is temperature, humidity, days of the week, season, holiday, and weekday. This shows us that the best subset of the feature depends on the data structure rather than the correlations of the features with the target variable. Thus, except exhaustive feature selection, the correlation analyses or any other method may lead astray during implementing feature-selection methods. Each feature can be significant for prediction accuracy, but the interactions of features may worsen the accuracy. Feature optimization also gives us a chance to study with a small subset of features. The optimization results in fast computing time and makes the data easily reachable. The simplicity of our model is an excellent advantage and also allows us to predict the patient arrivals without using any predicted value for hourly arrival rate data. For example, if we need weather features, then we will also need the prediction of weather variables of the year 2019 to predict the patient arrival rates of the year 2019. As a result, we do not need to estimate any feature to predict the patient arrival. In several studies that predict hourly arrival rates, the hour of the day has been the most important feature [4]. Until mean arrival rate feature is added, the day of the hour was a very important feature for us because, after adding the feature of mean arrival rate to the feature subset, the exhaustive feature-selection method was observed to eliminate the day of the hour from the best subset of features and select the mean arrival rate for the best subset of feature. Also, by using the feature of mean arrival rate, the model updated itself because mean arrival rate continuously updates itself as the time passes. The reviewed studies argue that temporal variables are more influential to patient arrivals than holiday and weather [1, 4, 37]. Our results indicate that weather variables do not affect the predictions of hourly patient arrivals. The reason of this can be the moderate climatic conditions in this region. This can be checked by performing this study in different regions with severe climatic conditions. In several studies, a higher number of patient arrivals at ED were observed to be on Mondays [1, 2, 4]. On weekends, the patient arrivals slightly increased [2, 14]. Our data analysis showed that the patient arrivals are nearly the same in any weekday, but at the weekend, the patient arrivals increase nearly by 25%. The significance of features was observed to be heavily dependent on the data structure.

In this study, for the same ED, the daily arrivals are predicted; hourly arrival rates are predicted both for one year and for five years. Using the five year dataset is one of the strengths of our model because this is one of the largest datasets among the studies that build an admission prediction model. Because multiple years of data would offer an advantage of more accurate predictions, there will be a chance to estimate discrepancies such as seasonal variation and to obtain more accurate values of mean arrival rate. For more complicated values, the LSTM model was observed to perform better than the other ML algorithms. Also, hourly prediction showed us that the ED faced several hours of crowding. The ED's decision-makers should focus on these hours, and resources should be planned by taking into consideration the overcrowding hours.

The RNN had several parameters; using trial and error, different number of hidden neurons, learning rate, and different activation functions were tested. By tuning these parameters, it is possible to improve the accuracy. But it is impossible to know whether the structure is optimal. The stateful LSTM model was observed to perform better than in other studies presented in the literature (e.g., [18]). The reason for this can be the architecture of the RNN model or the specific dataset we use. As demonstrated, the RF was observed to perform the best for the one-year data, but not for the five-year data.

In most studies, the LOS at the ED was observed to increase when hospital's bed occupancy exceeds 90% and strong negative correlation was observed between the patients who are waiting at the ED and the number of available beds (e.g., [17]). The relationship with the number of beds and overcrowding was easily demonstrated by the simulation models. Further focus should be laid on the busiest hours. LOS decreases as the number of beds increase from 7 to 10. However, increasing the number of beds beyond 10 does not lead to further decrease After this point, other resources, such as the staff, must be adjusted to decrease the LOS. As the number of beds increased, the bed queue decreased; however, after a certain threshold, there is no further improvement in queue. Hence, we found the optimum number of beds in our case to be 10. The solution we develop here has a potential to be used in practice because of its computing time, good performance, and simplicity. In the future, the solution can be incorporated to the hospital's management information system to adjust the resources of EDs on a periodic basis.

## 5. Conclusion

In this study, the potential features that have association with the number of patient arrivals were identified. Then, by using the exhaustive feature-selection method, the best subset of the feature that gives the most accurate predictions was determined. Ten ML algorithms were developed to predict the patients' arrival rates. The stateful LSTM was observed to perform the best, but the RF and the DT models also performed well. The predictions of patients' arrival rates were used as an input parameter for the simulation model that we developed. The route of the patients at the ED was extracted from the analysis of the hospital's data, and treatment/service times were determined from our observations with the help of the hospital senior management, doctors, and nurses. According to the results of our simulation, several resources are identified as bottlenecks in the ED. In this study, the main focus was on the number of beds. We used our simulation model with different number of beds, and the impact of the number of beds on the waiting time of patients at the ED was observed.

The RNN method is a black box approach that cannot interpret the relationship between input and output. To overcome this, a feature-selection approach was combined with the RNN model. However, feature-selection methods such as other wrapper approaches, filter methods, or correlation analyses may lead astray in feature-selection approaches. This is because the feature with the highest correlation with the target variable can worsen the accuracy of the prediction when used along with the combination of other features. Therefore, if allowed by the computing time constraints, a possible exhaustive feature-selection should be used to find the best subset for our prediction model. The importance of the features for the prediction model depends on the data structure and the used ML model.

Most of the studies in the literature used the fact that the prediction of patients' arrival rates to ED is a nonhomogeneous Poisson process with a rate *λ*(*t*). However, on the basis of MAPE, the mean arrival rate was observed to give more accurate results than those by nonhomogeneous Poisson processes.

In this study, it was easily observed that the performance of the ML algorithms depends heavily on the data structure. No algorithm was observed to work the best for every problem, and it was particularly relevant in the case of supervised learning (i.e., predictive modeling). For example, one cannot say that neural networks are always better than RF or vice versa. There are many factors at play, such as the size and structure of the dataset. As a result, one should try different algorithms for the problem, while using test set of data to evaluate the performance and select the model that performs the best. In light of these, ten ML algorithms were tested in this study.

The predictions of the stateful LSTM model were used as input to the simulation model that we developed. Using the simulation model, it was possible to observe the effect of the number of beds on the total LOS. Increasing the number of beds was observed to decrease the waiting time until a certain threshold. After that threshold, other resources must be optimized to further decrease the LOS. Hospital managers often find it difficult to plan and allocate resources efficiently on the expected patient inflow from the ED. This study shows that a decision tool can help to use resources efficiently, reduce overcrowding, and improve patient satisfaction. The main limitation of this study is that the data of the LOS at ED could not be reached.

The most important contribution of this study is using ML methods to generate input parameters (patient arrival rates) for a simulation model that is then used for capacity planning. In further studies, LOS can also be predicted by using ML methods. These predictions can be used as inputs to the simulation model leading to more reliable results. While performing future studies, the optimization should be carried out simultaneously considering all resources, rather than optimizing each resource independently. The approach of using ML's prediction outputs as the input parameters to a simulation model can be utilized in other healthcare management problems that require efficient resource utilization and are characterized by complex input and output relations.

## Figures and Tables

**Table 1:** Treatment/service times in each ED section.

Process	Distribution parameter	Source	Reference
Triage room	UNIF (2, 4)	Nurse	Hospital data
ED physician examination	TRIA (5, 8, 15)	ED physician	Hospital data
Lab and X-ray test	TRIA (15, 30, 45)	Laboratorian	Hospital data
Attending physician examination	TRIA (5, 10, 15)	Attending physician	Expert view
Patient stabilization	TRIA (30, 60, 120)	Bed	Expert view
Patient stabilization	TRIA (30, 60, 120)	Attending physician	Expert view
Pediatrician	TRIA (15, 20, 40)	Attending physician	Expert view
Otorhinolaryngologist	TRIA (5, 8, 20)	Attending physician	Expert view
Internal medicine specialist	TRIA (15, 20, 30)	Attending physician	Expert view
Orthopedics and traumatology specialist	TRIA (5, 10, 20)	Attending physician	Expert view

**Table 2:** Summary statistics of MAPE of 10 ML techniques across the 10-fold cross-validation, 1-year daily arrival data.

ML	LSTM	LR	DT	GBM	SG	AB	KNN	SVM	MLP	RF
Mean	16.79	20.83	26.10	20.19	21.04	22.24	20.54	21.57	21.81	21.88
SD	6.49	6.27	7.33	6.26	6.06	6.60	6.68	6.75	7.37	6.39
Min	11.78	14.18	14.28	13.37	14.51	13.75	13.94	14.38	14.05	13.20
Max	35.05	30.47	36.54	29.69	30.50	31.05	32.13	32.31	32.31	30.63

SD: standard deviation.

**Table 3:** Summary statistics of MAPE of 10 ML techniques across the 10-fold cross-validation, 1-year hourly arrival data.

ML	LSTM	LR	DT	GBM	SG	AB	KNN	SVM	MLP	RF
Mean	49.78	57.02	49.81	50.27	56.92	59.90	49.96	60.27	50.89	49.67
SD	5.64	2.14	2.45	4.14	2.20	2.68	2.30	2.57	3.09	2.45
Min	42.27	52.63	46.21	45.06	52.55	55.79	45.48	54.51	46.23	46.12
Max	57.46	61.34	55.68	56.14	61.31	65.09	55.00	64.96	58.38	55.58

SD: standard deviation.

**Table 4:** Summary statistics of MAPE of 10 ML techniques across the 10-fold cross-validation, 5-year hourly arrival data.

ML	LSTM	LR	DT	GBM	SG	AB	KNN	SVM	MLP	RF
Mean	46.73	59.21	50.77	52.68	59.08	79.12	52.19	96.17	52.12	50.78
SD	2.19	0.59	0.51	0.53	0.63	3.14	0.73	1.28	1.44	0.52
Min	42.99	58.18	50.00	51.61	58.04	71.14	51.06	94.31	50.08	49.99
Max	51.42	60.09	51.45	53.49	60.06	82.62	53.47	98.87	54.31	51.40

SD: standard deviation.

## Data Availability

The data used to support the findings of the study are available from the corresponding author upon request.

## Abbreviations

AB: AdaBoost
AI: Artificial intelligence
ABS: Agent-based simulation
ANN: Artificial neural network
ARIMA: Autoregressive integrated moving average
DES: Discrete-event simulation
DT: Decision tree
ED: Emergency department
GBM: Gradient boosted machines
KNN: K-nearest neighbors
LR: Logistic regression
LOS: Length of stay
LSTM: Long short-term memory
MAE: Mean absolute percentage error
ML: Machine learning
MLP: Multilayer perceptron
RF: Random forest
RNN: Recurrent neural network
SD: System dynamic
SG: Stochastic gradient regression
SVM: Support vector machine.
